# Extracellular matrix nitration alters growth factor release and activates bioactive complement in human retinal pigment epithelial cells

**DOI:** 10.1371/journal.pone.0177763

**Published:** 2017-05-15

**Authors:** Mark A. Fields, Hannah E. Bowrey, Jie Gong, Ernesto F. Moreira, Hui Cai, Lucian V. Del Priore

**Affiliations:** 1 Department of Ophthalmology and Visual Science, Yale School of Medicine, New Haven, CT, United States of America; 2 Brain Health Institute, Rutgers, The State University of New Jersey, Piscataway, NJ, United States of America; 3 Save Sight Institute, The University of Sydney, Sydney, NSW, Australia; 4 Department of Ophthalmology, Storm Eye Institute, Medical University of South Carolina, Charleston, SC, United States of America; Indiana University School of Medicine, UNITED STATES

## Abstract

**Purpose:**

We have shown previously that non-enzymatic nitration (NEN) of the extracellular matrix (ECM), which serves as a model of Bruch’s membrane (BM) aging, has a profound effect on the behavior of the overlying retinal pigment epithelial (RPE) cells, including altered phagocytic ability, reduced cell adhesion, and inhibition of proliferation. We know that transplanted RPE monolayers will encounter a hostile sub-RPE environment, including age-related alterations in BM that may compromise cell function and survival. Here we use our previous NEN model of BM aging to determine the effects of NEN of the ECM on growth factor release and complement activation in RPE cells.

**Methods:**

Human induced-pluripotent stem cells (iPSCs) were differentiated into RPE cells, and confirmed by immunohistochemistry, confocal microscopy, and polymerase chain reaction. IPSC-derived RPE cells were plated onto RPE-derived ECM under untreated or nitrite-modified conditions. Cells were cultured for 7 days and barrier function measured by transepithelial resistance (TER). Vascular endothelial growth factor (VEGF), pigment epithelium-derived factor (PEDF), and complement component C3a were measured using enzyme-linked immunosorbent assay (ELISA).

**Results:**

On average nitrite-modified ECM increased VEGF release both apically and basally by 0.15 ± 0.014 ng/mL (*p* <0.0001) and 0.21 ± 0.022 ng/mL (*p* <0.0001), respectively, in iPSC-derived RPE cells. Nitrite-modified ECM increased PEDF release in iPSC-derived RPE cells apically by 0.16 ± 0.031 ng/mL (*p* <0.0001), but not basally (0.27 ± 0.015 vs. 0.32 ± 0.029 ng/mL, (*p* >0.05)). Nitrite-modified ECM increased production of C3a in iPSC-derived RPE cells by 0.52 ± 0.123 ng/mL (*p* <0.05).

**Conclusion:**

Nitrite-modified ECM increased VEGF, PEDF release, and C3a production in human iPSC-derived RPE cells. This model demonstrates changes seen in the basement membrane can lead to alterations in the cell biology of the RPE cells that may be related to the development of age-related macular degeneration.

## Introduction

Alterations in the basement membrane are antecedent events in the development of numerous human disorders, including age-related degeneration (AMD), dystrophic epidermolysis bullosa, and Alport Syndrome [1–4]. Within the eye, aging of Bruch’s membrane (BM), whose innermost layer is the basement membrane of the retinal pigment epithelial (RPE) cells is an important and early step in AMD. In the development of disease, these alterations precede RPE changes by 1–2 decades and exert a deleterious effect on RPE cell behavior [3, 4]. While the exact age-related molecular changes that develop within BM are still being elucidated, we know that structural changes within BM include diffuse membrane thickening, accumulation of drusen, basal laminar and basal linear deposits [5, 6], collagen cross-linking in the inner and outer collagen layer, calcification and fragmentation of the elastin layer [7], and BM lipidization [7, 8]. The contribution of RPE cells to Bruch’s membrane health is significant. Large drusen that contribute to focal Bruch’s membrane thickening, arise partially from the RPE cells and contribute to aspects of AMD pathology such as choroidal neovascularization and geographic atrophy (GA) via cracks or loss of inner layers due to inadequate basal membrane regeneration [9, 10].

In homeostasis, RPE cell release of vascular endothelial growth factor (VEGF) and pigment epithelium-derived factor (PEDF) in a polarized fashion is an important regulator of complement activation [11, 12]. Because of the importance of these factors, and the known deleterious effects of Bruch’s membrane aging on RPE cell function [13], we attempt to investigate the effects of deleterious changes within the basement membrane on polarized release of VEGF, PEDF and production of the important complement component C3a [14]. To do this, we crosslink normal ECM using non-enzymatic nitration, which causes non-physiologic cross linking of proteins within the basement membrane and thereby mimics many of the effects of basement membrane aging that alter RPE cell behavior in vivo in elderly eyes with AMD.

We have shown that aging of human BM has a deleterious effect on critical cellular functions such as phagocytosis [13]. Collagen cross-linking of the extracellular matrix (ECM) derived from RPE cells serves as a relevant in vitro model of BM aging through non-enzymatic nitration of the basement membrane [13, 15]. This model has enabled the investigation of cellular behavior due to the age-related effects of BM substrate disease such as growth factor release and complement activation, and mimics the deleterious effects of substrate aging [16]. Here, we determine the effects of non-enzymatic nitration (NEN) of the ECM, which mimics the effects of BM aging on growth factor release as well as complement activation in an iPSC-derived RPE cell line.

VEGF is a critical survival factor for RPE cells, and its polarized release is required to stabilize the fenestrated structure of the endothelium of the choroid [17, 18]. The angiogenic effects of VEGF in the pathogenesis of “wet” or exudative AMD have been well-documented [19, 20], and patients with “dry” or non-exudative AMD can progress to the exudative form in some cases, suggesting an overlap in pathogenesis [21, 22]. Genome-wide meta-analysis studies have shown that the VEGF-A locus is associated with both exudative and non-exudative AMD and the multifactorial risk factors of the disease, such as oxidative stress and smoking, may contribute to both pathological forms [23–27]. Mice with increased VEGF-A levels exhibited both neovascular AMD and non-exudative AMD suggesting that VEGF dysfunction may play a role in both forms of the disease [28]. There is RPE cell and retinal dysfunction, as well as morphological abnormalities in mice, with increased VEGF-A at sites where no choroidal neovascularization (CNV) lesions appear to be present, thus supporting a role for this protein in the non-exudative form of AMD [21]. PEDF is an endogenous growth factor secreted by RPE cells that has neuroprotective, neurotrophic, and antiangiogenic activity [29, 30]. PEDF acts as a counterbalance to the effects of VEGF and is thought to help maintain the integrity of the RPE cell barrier [31–33].

Activation of the complement system has been shown to be a major contributor to the pathogenesis of AMD and is a reflection of the chronic inflammation seen at the site of disease [34–39]. Interestingly, bioactive complement components such as C5a and C3a have been identified in the drusen of patients with AMD, suggesting complement activation in the area of RPE cells and BM [14]. Both forms have been shown to induce VEGF production in RPE cells in vitro [14]. Activation of C3a has also been demonstrated in in vitro and in vivo models of smoke exposure and oxidative stress, and a key factor in the development of AMD [40–44]. Accumulation of these factors and local inflammation may contribute to the pathogenesis of this disease [45].

## Materials and methods

### Ethics statement

All experiments were conducted with the approval of the Institutional Review Board (IRB # Pro00023262) and after written informed consent by study participants. This work was performed in adherence to the tenets of the Declaration of Helsinki.

### Human iPSC culture

Human iPSCs were derived from fibroblasts of a 71-year-old female donor (donor 1) with no history of retinal disease and a 72-year-old female with GA secondary to AMD (donor 2). Skin specimens from punch biopsies were transplanted onto culture plates and incubated for 1 hour with 0.5% dispase (Invitrogen-Gibco, Life Technologies, Grand Island, NY) and phosphate-buffered saline (PBS) in a 37°C water bath, then separated as an intact sheet by gentle agitation or by using two forceps. Cells were then placed in Dulbecco’s Modified Eagle’s Medium (DMEM; Invitrogen-Gibco, Life Technologies) and cultured in a humidified 37°C, 5% CO_2_ incubator until fibroblast outgrowth. Fibroblasts were grown to confluence and then treated with four Yamanaka factors, Oct3/4, Sox-2, Klf4, and c-Myc using the CytoTune™-iPS 2.0 Sendai Reprogramming Kit according to the manufacturer’s instructions (Invitrogen-Gibco, Life Technologies) [46–51]. Newly generated iPSC colonies were purified and expanded using MTeSR™1 media (Stem Cell Technologies, Vancouver, Canada). Cell numbers were expanded by passaging every 5–7 days using Accutase (Sigma-Aldrich) and cultured for use in downstream experiments.

### Differentiation of human iPSCs into RPE cells

Human iPSCs were differentiated to iPSC-derived RPE cells using a modified protocol previously described [52–54]. Human iPSC colonies were lifted with Accutase (1 mg/mL) and grown as embryoid bodies (EBs) by using AggreWell^TM^ 400 plates (STEMCELL Technologies, Vancouver, Canada) for 4 days in EB formation medium (STEMCELL Technologies). After 5 days, EB medium was replaced with neural induction medium (NIM) containing DMEM/F12 (1:1), 1% N-2 supplement (MEM non-essential amino acids and 2 μg/mL heparin). After 7 days, suspended EB aggregates were plated onto laminin-coated culture plates and allowed to reattach. Cells were grown for an additional 10 days in neural induction medium. After 16 days, neural induction medium was replaced with retinal differentiation medium (RDM) containing DMEM/F12 (3:1), 2% B-27 supplement (Invitrogen-Gibco, Life Technologies), MEM non-essential amino acids and penicillin/streptomycin. The adherent culture was maintained in RDM until the appearance of pigmented iPSC-derived RPE cells. Patches of pigmented iPSC-derived RPE cells were then micro-dissected, dissociated with trypsin-ethylenediaminetetraacetic acid (EDTA, 0.05%) and plated onto laminin-coated Transwell^®^ permeable inserts (Corning Inc., 3460-Clear, 0.4 mm pores, 12 mm inner diameter, polyester membranes). IPSC-derived RPE cells were cultured on Transwell^®^ plates with RDM + 10% fetal bovine serum (FBS) for 2 days and then switched to RDM with 2% FBS until the cells were confluent. Human iPSC-derived RPE cells were then maintained in RDM and allowed to form monolayers and re-pigment within 60–90 days.

### Preparation of ECM

Immortalized human RPE (ARPE-19) cells were obtained from the ATCC, cultured, and maintained in DMEM (Invitrogen-Gibco, Life Technologies) containing 10% FBS, 100 IU/mL penicillin, 100 μg/mL streptomycin, and 100 μg/mL gentamicin (Invitrogen-Gibco, Life Technologies). These cells were incubated in a humidified atmosphere of 5% CO_2_ and 95% air at 37°C. RPE cell-derived ECM (RPE-ECM) nitrite-treated plates were prepared as previously described [13, 55]. ARPE-19 cells were grown on 24-well Transwell^®^ permeable supports (Corning, Inc.) in 12-well plates or flat bottom 24-well plates for 6–8 weeks to allow the ECM to form. ARPE-19 cells were then removed by the addition of 20 mM ammonium hydroxide buffer for 20 min, and the ECM was washed with PBS. PBS was removed from the RPE-ECM plates and dried. These plates were used to create two experimental plating surfaces (untreated ECM and nitrite-modified ECM).

### Nitrite modification of the ECM

Nitrite-modified ECM was prepared as previously described [13, 15, 55, 56]. Briefly, 100 mM sodium nitrite was added to the ECM and incubated at 37°C for 7 days. Plates were then washed with PBS and incubated with PBS for 4 h. Finally, plates were washed with PBS to completely remove the nitrite.

### Immunohistochemistry

Cells were fixed with 4% paraformaldehyde at 4°C, then permeabilized with 0.1% Triton X-100 in PBS and incubated with 0.1% bovine serum albumin or 1% normal goat serum in PBS. Cells were incubated with various primary monoclonal or polyclonal antibodies for 2–3 h at room temperature, or overnight at 4°C. Cells were washed and incubated for 1 h at 37°C in the dark with rabbit anti-mouse or goat anti-rabbit IgG antibodies conjugated to either Alexa TM 594 (red fluorescence) or Alexa TM 488 (green fluorescence) (Invitrogen-Molecular Probes). Nuclei were stained with diamidinophenyl indole (DAPI, Sigma) for 5–10 minutes. A summary of antibodies used in this study is provided in Table 1. Immunologically-stained cell cultures were visualized by a Zeiss 510 NLO confocal laser scanning microscope using a Plan-Apochromatic 20x0.8 DIC objective.

**Table 1:** Antibodies used for staining target cells.

Antibody	Company	Dilution	Target Cells
**OCT-4**	EMD Millipore, Temecula CA	1:250	pluripotent stem cell
**Sox-2**	EMD Millipore, Temecula CA	1:100	pluripotent stem cell
**Nanog**	EMD Millipore, Temecula CA	1:100	pluripotent stem cell
**TRA-1-60**	EMD Millipore, Temecula CA	1:200	pluripotent stem cell
**ZO-1**	Invitrogen-Gibco, Life Technologies, Grand Island, NY	1:100	RPE cell (junction)
**Na + /K + ATPase**	EMD Millipore, Temecula CA	1:100	RPE cell
**RPE65**	Novus Biologicals, Littleton, CO	1:100	RPE cell
**Bestrophin**	EMD Millipore, Temecula CA	1:100	RPE cell

OCT-4, octamer-binding transcription factor-4; Sox-2, SRY (sex determining region Y)-box 2; Nanog, Nanog homeobox; TRA-1-60, podocalyxin; ZO-1, zonula occludens 1; RPE65, Retinal pigment epithelium-specific 65 kDa protein; Na + /K + ATPase, sodium-potassium adenosine triphosphatase; RPE cell, retinal pigment epithelial cell

### Transepithelial resistance

Human iPSC-derived RPE cells were plated onto one of two ECM conditions (untreated ECM and nitrite-modified ECM) in Transwell^®^ permeable supports (Corning, Inc.) containing RPE-ECM as described above. Cells were grown to confluence for one week in RDM containing 2% FBS. To confirm confluence, barrier function of iPSC-derived RPE cell monolayers was assessed by monitoring transepithelial resistance (TER) using an epithelial volt/ohm meter and an electrode (STX2; World Precision Instruments, Sarasota, FL) on RPE cell-derived ECM. The resistance values (Ω*cm^2^) were determined from the average of four independent measurements, and corrected for background resistance produced by the insert in the presence of RPE cell-ECM and media [33, 57].

### VEGF/PEDF/C3a assays on nitrite-modified ECM

Human iPSC-derived RPE cells were either plated onto untreated ECM or nitrite-modified ECM as described above. Cells were grown to confluence in RDM containing 2% FBS. Media was removed and the cells were washed with PBS. Serum-free RDM was added to cultured human iPSC-derived RPE cell monolayers for 24 h before conducting experiments. The supernatants were then collected and assayed for VEGF and PEDF using a human VEGF Quantikine ELISA kit (R&D Systems, Minneapolis, MN) or human PEDF ELISA kit (BioProductsMD, Middletown, MD), respectively. Supernatants were also collected and assayed from flat bottom 24-well plates for C3a using a human C3a ELISA kit (BD Biosciences, San Jose, CA). The results were analyzed using a SpectraMax M5 Multi-Mode Microplate Reader (Molecular Devices, Inc., Sunnyvale, CA) set at 450 nm. Aliquots were assayed in triplicate and values were compared with a VEGF_165_, C3a-des Arg, or human PEDF antigen standard curve.

### Data analysis

All data were analyzed with the average of two samples, and conducted in triplicate for each experiment. Independent, two-tailed *t* tests were performed using Prism (GraphPad Software, Inc., La Jolla, CA). A criterion of α = 0.05 was adopted.

## Results

### Human iPSCs differentiate into RPE cells and express RPE markers

**Fig 1:**
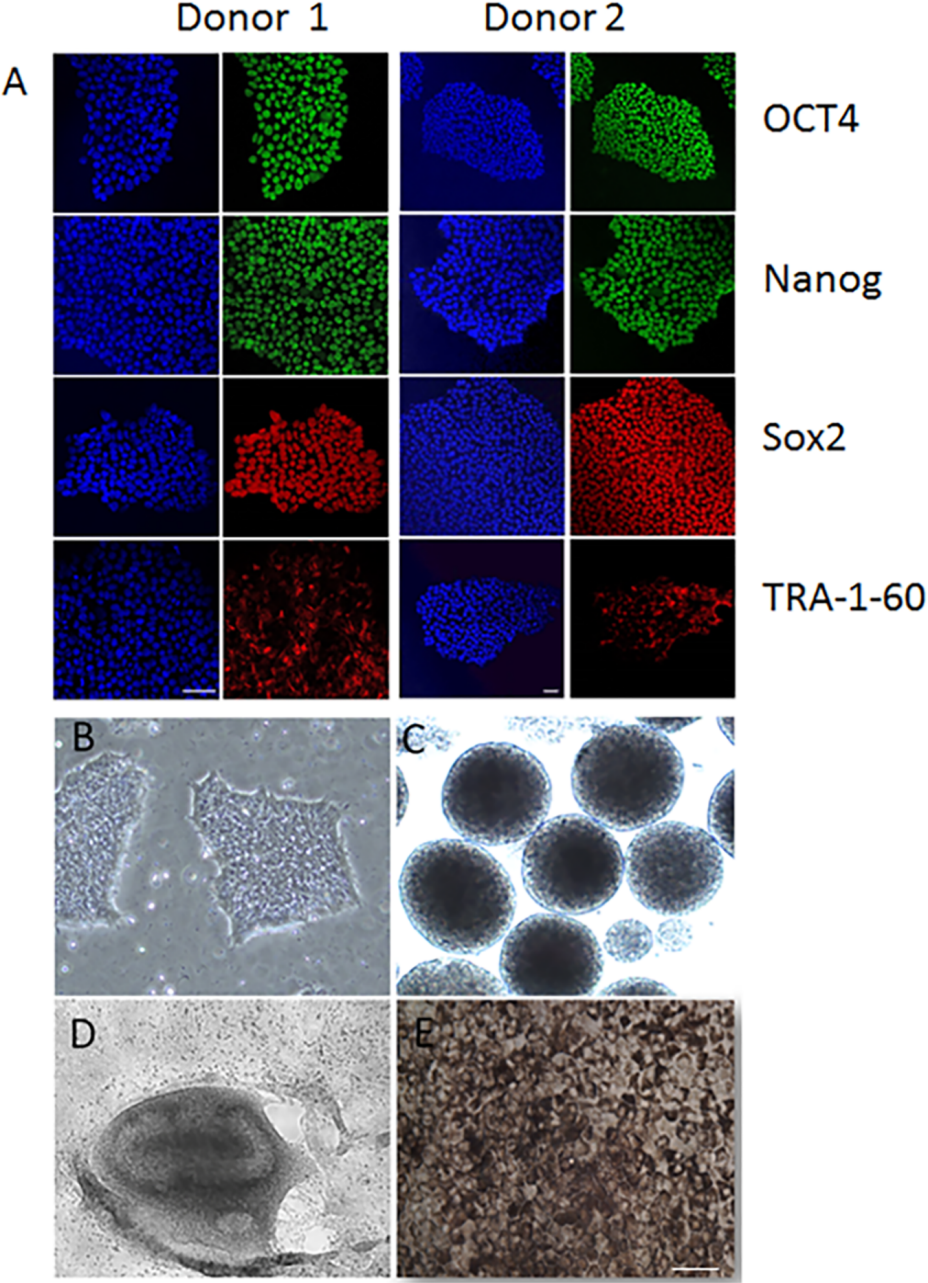
Differentiation of human induced-pluripotent stem cells (iPSCs) into iPSC-derived retinal pigment epithelial (RPE) cells from 2 different donors. Immunofluorescent staining was positive for Oct-4, Nanog, Sox-2, and TRA-1-60 (**A**). Nuclei stained with DAPI (blue). Undifferentiated human iPSC colony (**B**) and eventual formation into embryoid bodies (**C**). Formation of neural rosettes by day 14 post-differentiation (**D**), and a pigmented monolayer of iPSC-derived RPE cells forms by day 45 post-differentiation (**E**). Scale bar for **A** and **E** = 50 μm.

**Fig 2:**
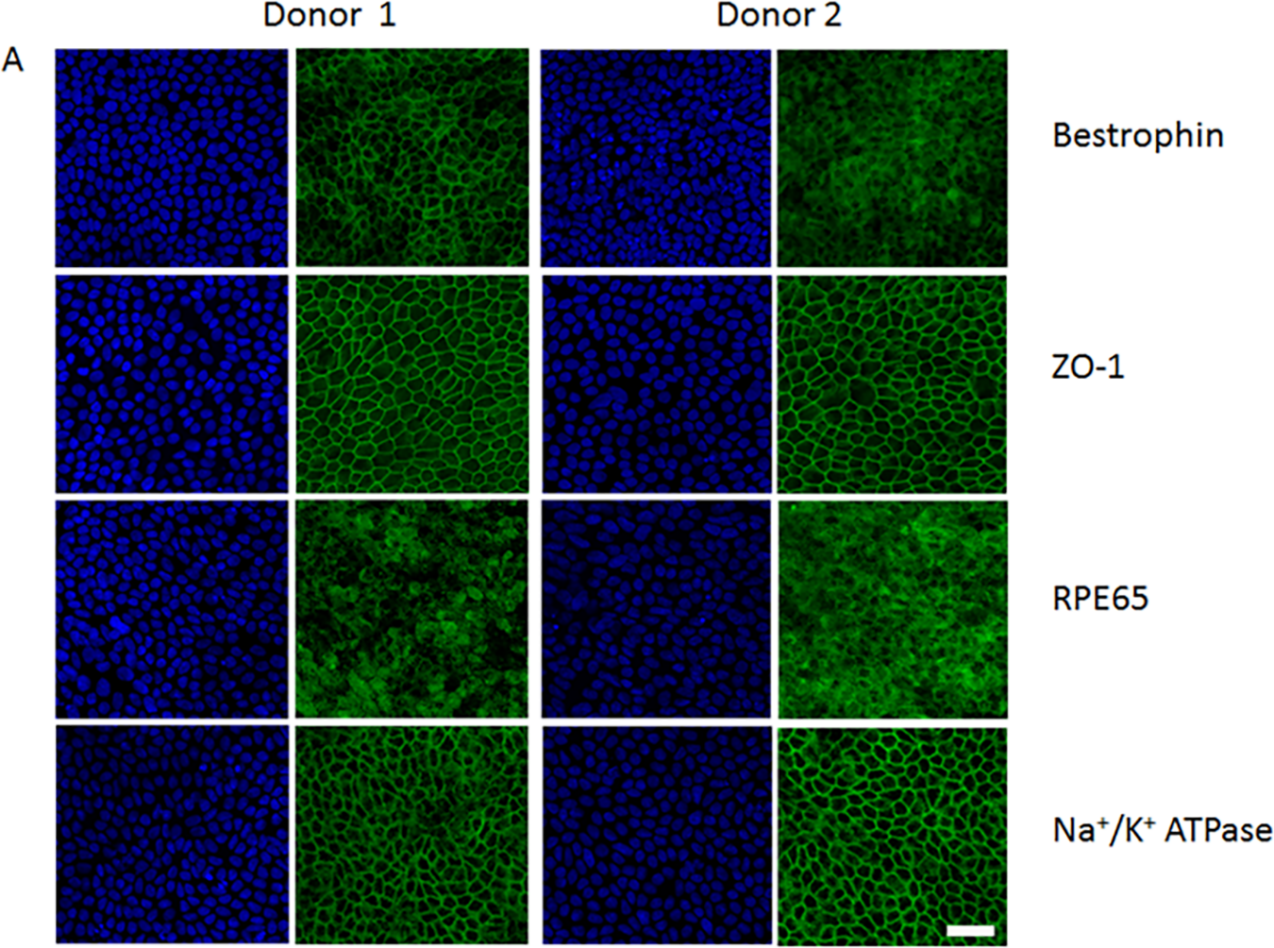
Expression and localization of retinal pigment epithelial (RPE) cell markers in human iPSC-derived RPE. Immunofluorescent staining was positive for bestrophin, Na + /K + ATPase, RPE65, and ZO-1 (**A**). Nuclei stained with DAPI (blue). Scale bar for **A** = 50 μm.

After Sendai virus transduction of octamer-binding transcription factor 3/4 (Oct3/4), (sex determining region Y)-box 2 (Sox-2), Kruppel-like factor 4 (Klf4), and regulator gene Myc that codes for a transcription factor (c-Myc) the cells expressed four markers (Fig 1A), octamer-binding transcription factor 4 (OCT-4), Nanog homeobox (Nanog), SRY (sex determining region Y)-box 2 (Sox-2), and podocalyxin (TRA-1-60), indicating these cells were pluripotent. To obtain human iPSC-derived RPE cells, iPSCs (Fig 1B) were induced to generate embryoid bodies (Fig 1C) and differentiated using a modified protocol described previously toward an RPE cell fate displaying neural rosettes (Fig 1D) [52–54]. After 30 days of differentiation, RPE-like cells were visible, expressing a hexagonal monolayer of RPE cell phenotype, and were purified (Fig 1E). After 45 days, differentiated iPSC-derived RPE cells robustly expressed bestrophin, the tight junction marker ZO-1, RPE65, and Na + /K + ATPase (Fig 2).

### ECM nitrite modification increased VEGF production in iPSC-derived RPE cells

**Fig 3:**
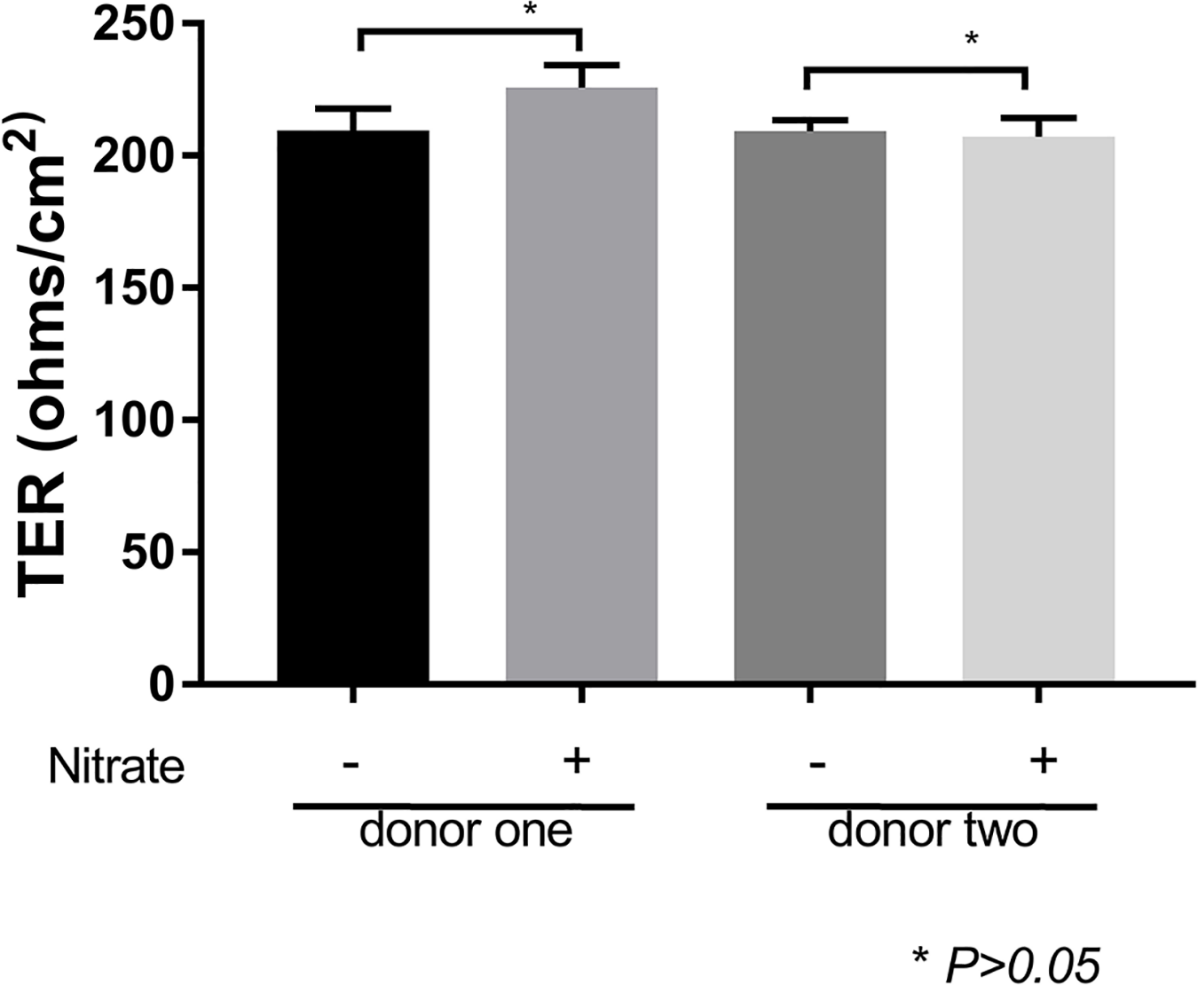
Transepithelial resistance (TER) of iPSC-derived RPE cells on RPE cell-derived ECM and nitrite-modified RPE cell-derived ECM. Monolayer permeability was assessed by TER in polarized iPSC-RPE cells. TER measurements were 209.5 and 209.3 Ω*cm^2^ for iPSC-derived RPE cells cultured on untreated extracellular matrix. iPSC-derived RPE cells cultured on nitrite-modified ECM were (225.8 and 207.3 Ω*cm^2^) from donor one and two samples, respectively.

**Fig 4:**
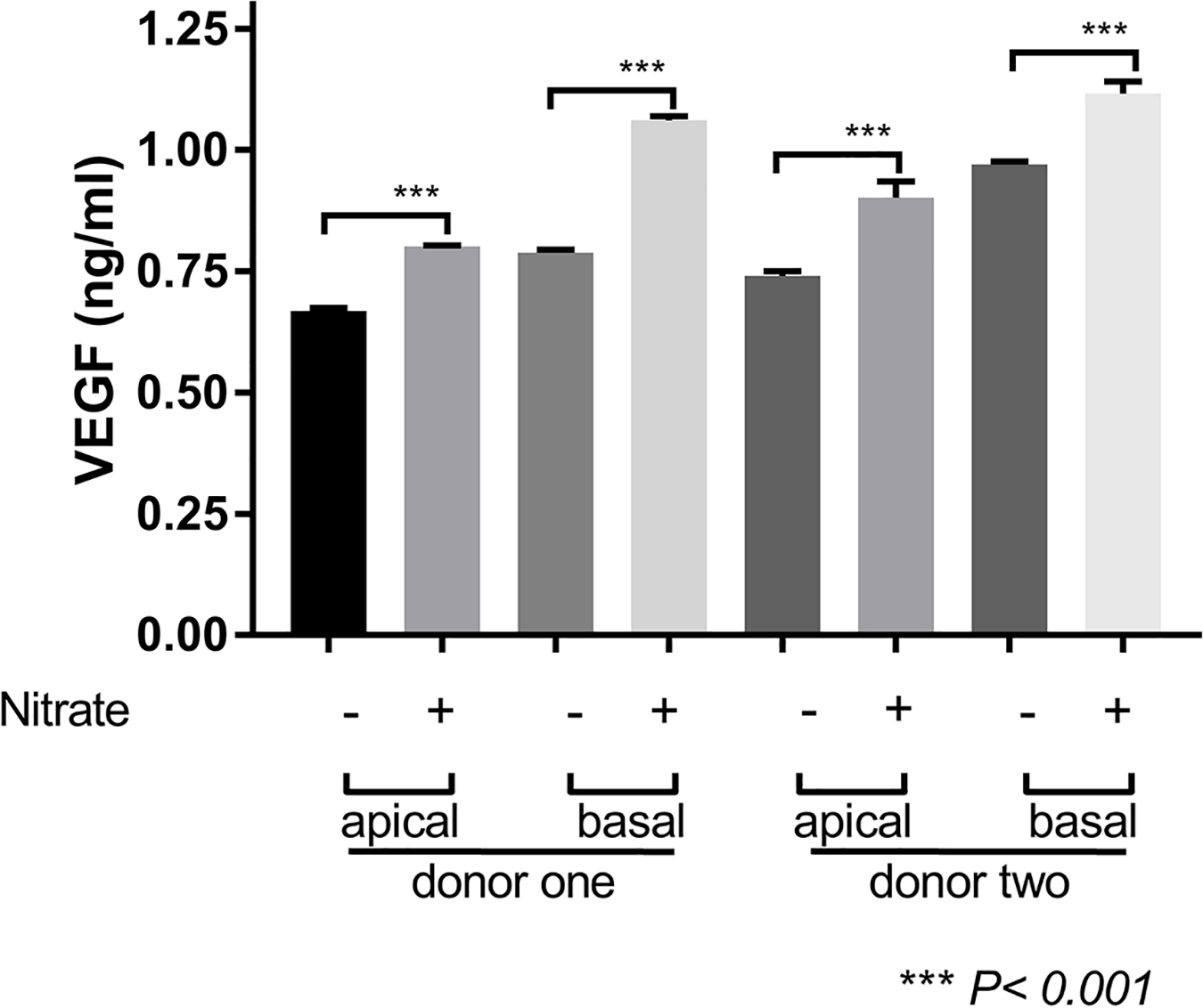
VEGF release in iPSC-derived RPE cell monolayers on RPE cell-derived ECM and nitrite-modified RPE cell-derived ECM. iPSC-derived RPE cells release of vascular endothelial growth factor (VEGF) on nitrite-modified ECM (nitrite) and untreated ECM (PBS) both apically and basally. ****p <0.0001.

In the following experiments we used a previously established approach of nitrite-modification of RPE cell-derived ECM to model the “aging” effect on BM [15]. The transepithelial resistance values for iPSC-derived RPE cells seeded on untreated ECM were 209.5 ± 8.4 and 209.3 ± 4.15 Ω*cm^2^, and nitrite-modified ECM were 225.8 ± 8.5 and 207.3 ± 7.0 Ω*cm^2^ from two donor samples, respectively (*p* >0.05; Fig 3). The data were within reported experimental range based on TER studies of ex vivo RPE cell monolayers [58, 59]. We then investigated the release of VEGF on these surfaces. Nitrite modification increased the apical release of VEGF from iPSC-derived RPE cells (0.6679 ± 0.067 vs. 0.8017 ± 0.0151 ng/mL in donor one, and 0.7409 ± 0.0089 vs.0.9023 ± 0.0330 in donor two, respectively, *p* <0.0001; Fig 4). Additionally, the basal release of VEGF was lower in iPSC-derived RPE cells seeded onto untreated ECM, relative to nitrite-modified ECM (0.7886 ± 0.063 vs. 1.061 ± 0.0091 ng/mL in donor one, and 0.9696 ± 0.0075 vs. 1.116 ± 0.0254 in donor two, respectively, *p* <0.001; Fig 4). Thus relative to the untreated ECM, nitrite modification of the ECM surface led to increased levels of apical and basal VEGF in iPSC-derived RPE cells.

### ECM nitrite modification increased PEDF release in iPSC-derived RPE cells

**Fig 5:**
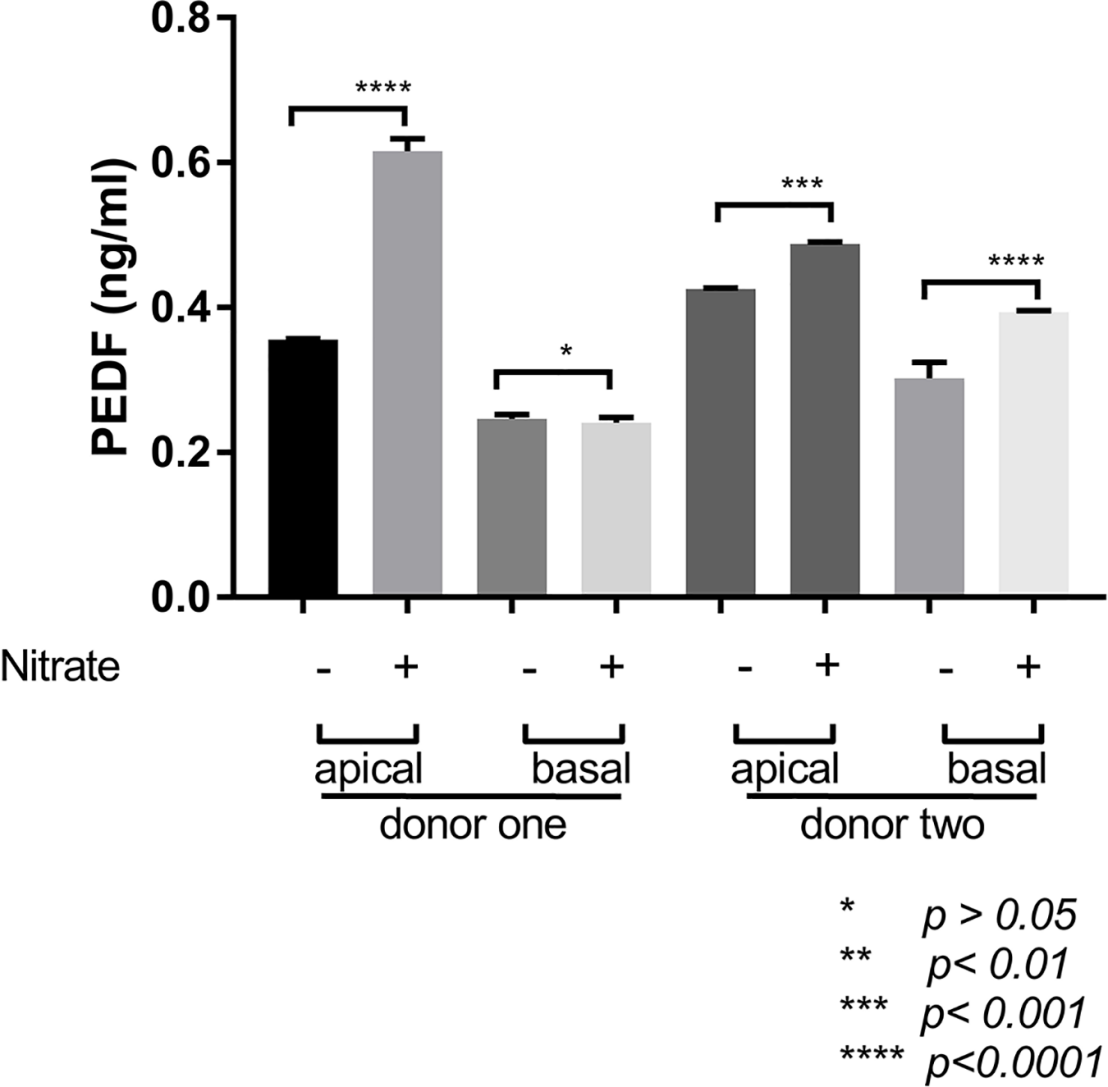
PEDF release in iPSC-derived RPE cell monolayers on RPE cell-derived ECM and nitrite-modified RPE cell-derived ECM. iPSC-derived RPE cell release of pigment epithelium-derived factor (PEDF) increased on nitrite-modified ECM (nitrite) apically. **** *p* <0.001.

The apical release of PEDF was lower in iPSC-derived RPE cells seeded onto untreated ECM, relative to nitrite-modified ECM (0.3555 ± 0.0017 vs. 0.616 ± 0.0171 ng/mL in donor one, and 0.4255 ± 0.0013 vs. 0.488 ± 0.0021 in donor two, respectively, *p* <0.001; Fig 5). The basal release of PEDF from the overlying iPSC-derived RPE cells on untreated and nitrite-modified ECM samples was 0.2463 ± 0.0062 vs. 0.2413 ± 0.0070 in donor one (*p* >0.05), and 0.3023 ± 0.0223 vs. 0.3938 ± 0.0022 in donor two (*p* <0.001), respectively, (Fig 5). Thus, relative to the untreated ECM, nitrite modification of the ECM surface led to increased levels of PEDF in iPSC-derived RPE cells, apically, but not basally.

### ECM nitrite modification increased C3a production in iPSC-derived RPE cells

**Fig 6:**
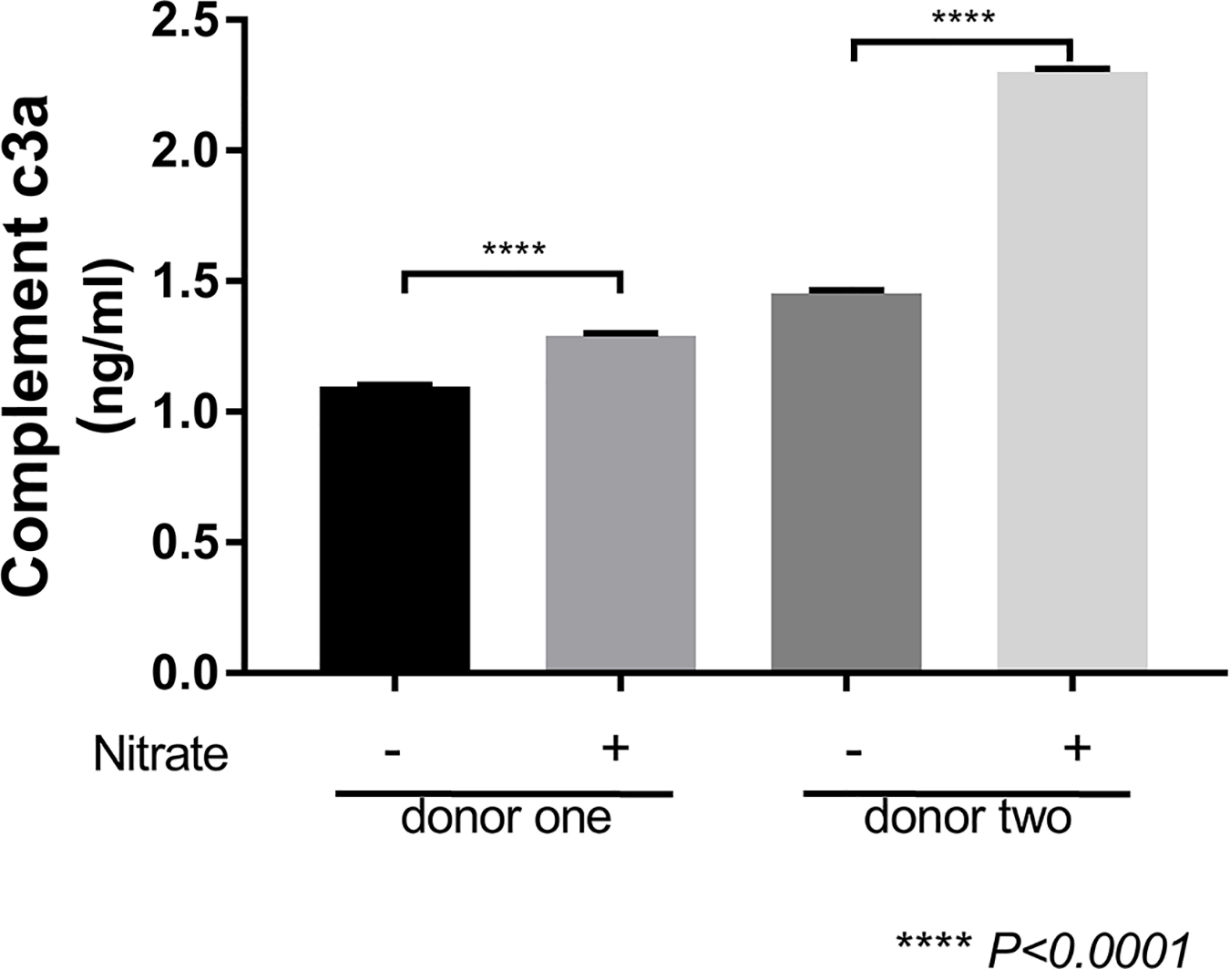
C3a production in iPSC-derived RPE cell monolayers on RPE cell-derived ECM and nitrite-modified RPE cell-derived ECM. iPSC-derived RPE cell C3a production increased on nitrite-modified ECM (nitrite). * *p* <0.0001.

The release of C3a was lower in iPSC-derived RPE cells seeded onto untreated ECM, relative to nitrite-modified ECM (1.096 ± 0.0063 vs. 1.29 ± 0.0102 ng/mL in donor one, and 1.453 ± 0.127 vs. 2.301 ± 0.0135 in donor two, respectively, *p* <0.001; Fig 6). Therefore, relative to untreated ECM, nitrite modification of the ECM surface led to increased production of C3a in iPSC-derived RPE cells.

## Discussion

We used an in vitro model of BM “aging” to investigate cellular release of VEGF, PEDF, and production of C3a in iPSC-derived RPE cells. We demonstrated that the behavior of RPE cells differs when the ECM is nitrite-modified, a process that occurs during chronic inflammation and in the presence of risk factors associated with AMD such as cigarette smoking [15, 35–37, 60–64]. These findings are in line with previous studies in ARPE-19 and primary calf RPE cell cultures that demonstrate nitrite modification of the ECM affects normal RPE cell behavior, such as attachment, proliferation, phagocytic ability and susceptibility to light-mediated damage by A2E [13, 15, 65].

Here we test similar parameters in therapeutically relevant cell lines generated from skin biopsies of older individuals with and without AMD [66, 67]. Using these iPS-derived RPE cells we demonstrated that secreted VEGF and PEDF levels were increased on nitrite-modified ECM (Figs 4 and 5). VEGF levels were increased both apically and basally in iPSC-derived RPE cells while PEDF levels were increased apically alone; there was no difference in the release pattern of PEDF basally on nitrite-modified ECM when compared to the untreated ECM when combining two donor samples together for statistical analysis. Consistent with past reports, the PEDF pattern of release is higher on the apical side in established monolayers of RPE cells [33]. Alternatively, VEGF production is typically higher on the basal side of established RPE cell monolayers, and amounts increase under stress such as hypoxic conditions in vitro [18]. While the release of PEDF increased apically, it did not exceed that of VEGF on the nitrite-modified ECM. Moreover, the basal release of PEDF did not change with nitrite modification of the surface, despite increased basal VEGF release. This suggests that the VEGF-PEDF ratio ultimately favored VEGF release after nitrite modification, which may favor progression to angiogenesis [68].

From a pathological standpoint, angiogenesis is a complex biological event that involves a balance between stimulators and inhibitors such as VEGF and PEDF. VEGF and PEDF have been shown to be expressed in the RPE cell-Bruch's membrane-choriocapillaris tissue complex in aged subjects [69]. It has been reported that VEGF increases the expression of PEDF by human RPE cells in a physiologic state [70]. The increased apical release of PEDF may suggest a regulatory role for the inhibitor in this model. There is evidence to suggest that endogenous antiangiogenic factors like PEDF maintain a vascular quiescence that is otherwise disturbed by factors such as VEGF [69]. Moreover, it has been demonstrated that PEDF is a major angiogenic inhibitor in the eye and may have a protective role against oxidative stress [30, 71, 72]. Our data suggest that in an environment of enhanced or altered VEGF release, PEDF may be acting as a counterbalance to this phenomenon. While these results are intriguing, more studies in therapeutically relevant cell lines remain to investigate the ratio of VEGF-PEDF release in this model and its relationship to RPE cell stress and BM aging.

We have also demonstrated that the production of C3a by iPSC-derived RPE cells is increased on the nitrite-modified ECM. Increased complement activation has been associated with disease progression in AMD [14, 73, 74]. This activation can lead to pro-inflammatory damage to the RPE cell-BM-choriocapillaris tissue complex, and continued damage that can potentially lead to neovascularization manifested as CNV or the atrophic damage manifested as geographic atrophy (GA) in late stage AMD [75, 76]. Moreover, complement components, such as C3a and C5a, have been identified in the drusen of AMD patients [14, 77]. Our observation that nitrite modification of the ECM increased C3a production, VEGF release, and apical PEDF release would suggest a relationship between the structural changes seen in aged or diseased BM and the cellular changes (atrophy and neovascularization) in RPE cells seen during the development of AMD.

C3a is the bioactive form of C3 and there is growing evidence of its role in AMD. Nozaki et al. observed the presence of C3a in drusen deposits of patients with AMD, suggesting that RPE cells are exposed to this inflammatory protein in this state [14]. Treatment with C3a protein both in vitro and in vivo has also been shown to induce VEGF in RPE cells and wild-type C57BL/6J mice, respectively [14]. Our data suggest that nitrite modification of the ECM plays a role in inducing C3a production in iPSC-derived RPE cells and may contribute to the release of VEGF in these cells. The relationship between complement activation and pro-inflammatory mediators in AMD continues to be investigated [75]. Models that mimic BM aging by treatment of the ECM with sodium nitrite, a nonenzymatic crosslinking agent, can help elucidate these disease mechanisms [13, 65]. While there appears to be a difference in the donor with GA in terms of VEGF and PEDF release as well as C3a production, we feel that the sample size is insufficient to decipher whether this is due to a disease state or other variables. In the future, we will address this by increasing the number of cell lines to draw valid conclusions between disease and non-disease states.

**Fig 7:**
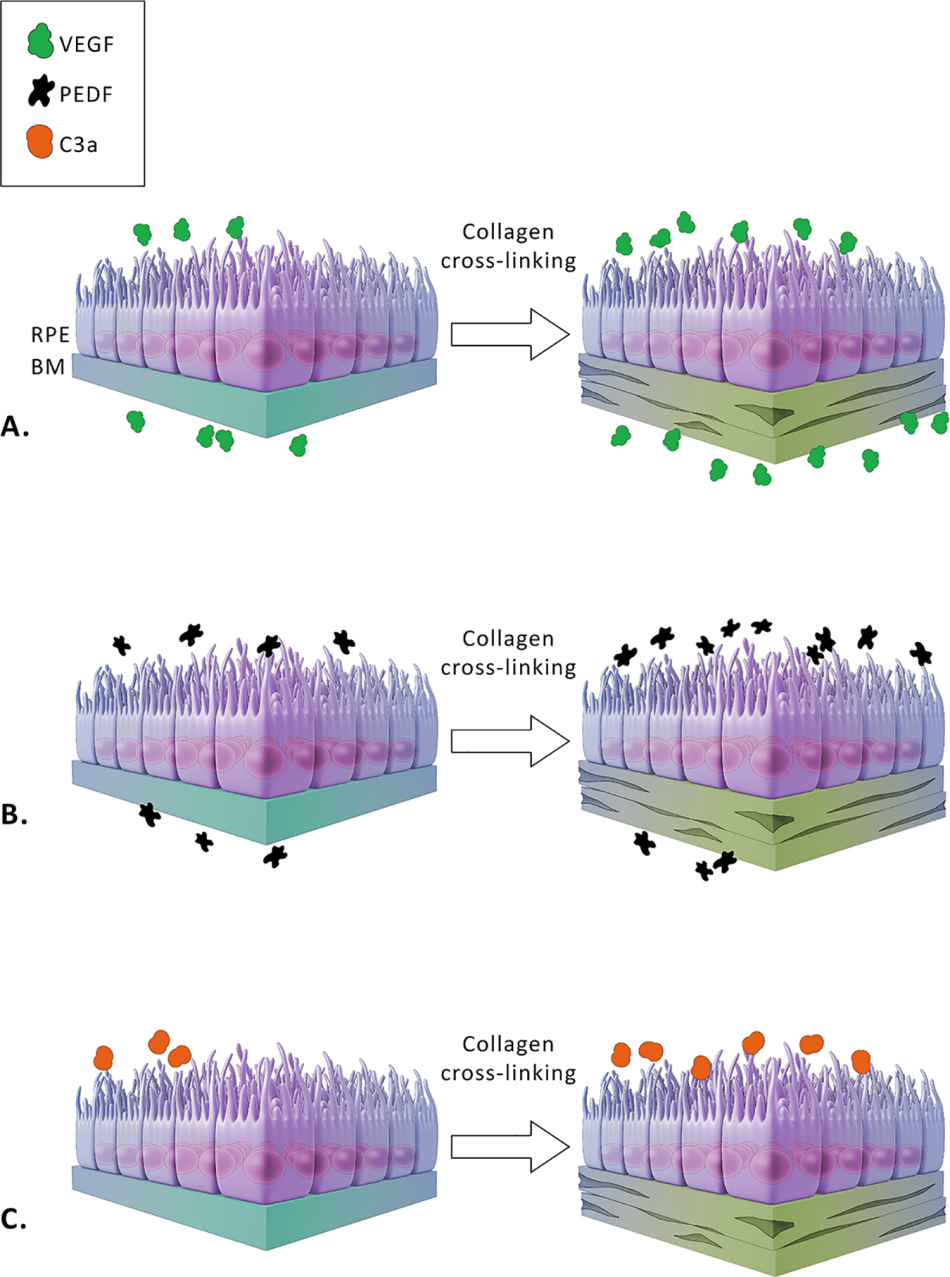
Effects of nitrite modification on VEGF, PEDF and C3a in iPSC-derived RPE cells. Release of VEGF, PEDF and c3a from patient-derived iPSC-RPE cells on normal versus “aged” extracellular matrix is represented diagrammatically. VEGF release is higher basally than apically on normal basement membrane (**A**). Cross-linking of collagen in the basement membrane increased release of VEGF both apically and basally in iPSC-derived RPE cells while preserving this polarity (**A**). PEDF release is higher apically than basally on normal basement membrane (**B**). Cross-linking of collagen in the basement membrane increased release of PEDF apically, and preserved this polarity (**B**). Cross-linking of collagen in the basement membrane increased release of C3a in iPSC-derived RPE cells (**C**). The aging of Bruch’s membrane increases VEGF (apical and basal) and PEDF (apical and basal) release in the RPE cells as well as C3a production. Increased release of these factors may contribute to the pathology at the site of RPE cells seen in exudative and advanced dry age-related macular degeneration (AMD) or geographic atrophy.

Our data add further support to the understanding that alterations in the basement membrane, an important portion of BM, can induce changes in the overlying RPE cells. Fig 7 summarizes the effects of basement membrane alterations on the overlying RPE cells as seen in this study. Aging is the most significant risk factor for the development of AMD, and BM undergoes major changes during this process [42]. As mentioned, these changes include drusen formation, development of basal laminar and basal linear deposits [5, 6], collagen cross-linking of the collagen layers, calcification and fragmentation of the elastin layer [7], and membrane lipidization [7, 78, 79]. Other key elements contributing to the structural changes in BM are exposure to nitrite during chronic inflammation from aging and smoking [15, 80]. Our data suggest that treatments for AMD will certainly need to consider the state of BM. Models of retinal degenerative disease such as those of ‘aging’ could prove valuable to study complex diseases such as AMD that include both the contribution of genetic mutation and environmental risk factors [66, 81, 82].

## Conclusion

In order to completely understand AMD pathophysiology, and for cell-based therapies for AMD to be viable, the issue of BM damage must be addressed. This study demonstrates that nitrite modification of the ECM increased VEGF and PEDF release in iPSC-derived RPE cells cultured onto this surface. Nitrite modification also increased the production of C3a in iPSC-derived RPE cells. This approach may be beneficial as a tool for disease modeling by mimicking the structural changes on BM and observing iPSC-derived RPE cell behavior on this surface [13, 83].

## Abbreviations

AMD: age-related macular degeneration
RPE: retinal pigment epithelial cells
BM: Bruch’s membrane
iPSC: induced pluripotent stem cell
VEGF: vascular endothelial growth factor
PEDF: pigment epithelium-derived factor
ZO-1: zonula occludens protein-1
RPE65: retinal pigment epithelium specific protein-65
MITF: microphthalmia-associated transcription factor
EBs: embryoid bodies
GA: geographic atrophy
CNV: choroidal neovascularization
ECM: extracellular matrix
CRALBP: cellular retinaldehyde-binding protein
NEN: non-enzymatic nitration
Oct3/4: octamer-binding transcription factor 3/4
Klf4: Kruppel-like factor 4
c-Myc: regulator gene that codes for a transcription factor Myc
Nanog: Nanog homeobox
TRA-1-60: podocalyxin
Sox-2: SRY (sex determining region Y)-box 2
Oct-4: octamer-binding transcription factor 4
